# Allergens of Regional Importance in Korea

**DOI:** 10.3389/falgy.2021.652275

**Published:** 2021-03-05

**Authors:** Kyoung Yong Jeong, Jung-Won Park

**Affiliations:** Department of Internal Medicine, Institute of Allergy, Yonsei University College of Medicine, Seoul, South Korea

**Keywords:** native species, invasive species, allergen repertoire, allergen, allergy diagnosis

## Abstract

Allergen repertoire should reflect the region's climate, flora, and dining culture to allow for a better diagnosis. In Korea, tree pollens of oak and birch in the spring in conjunction with weed pollens of mugwort, ragweed, and Japanese hop are the main causes of seasonal allergic rhinitis. More specifically, the sawtooth oak in Korea and the Japanese hop in East Asia make a difference from western countries. Among food allergens, the sensitization to silkworm pupa and buckwheat is also common in Korean patients. Honey bee venom due to apitherapy in traditional medicine and Asian needle ant, *Pachycondyla chinensis*, are important causes of anaphylaxis in Korea. Climate change, frequent overseas traveling, and international product exchanges make situations more complicated. Ragweed, for example, was not native to Korea, but invaded the country in the early 1950s. Recently, Japanese hop and Asian needle ants have been recognized as important invasive ecosystem disturbing species in western countries. However, the molecular properties of the component allergens from these unique culprit allergens have been poorly characterized. The present review summarizes the molecular studies on the allergens of regional importance in Korea.

## Introduction

Sensitization to an allergen should reflect the exposure from the allergen in the environment. The environment includes climate, which determines fauna and flora, and cultural differences including dining. Different plant and animal species produce different allergen molecules. Some allergens, especially for molecules called pan-allergens, share highly conserved IgE epitopes. However, several differences exist even between allergens that belong to the same protein family. The same species can also produce different isoallergens or isoforms. Different food processing, which reflects cultural aspects, also affects the integrity and modifies the allergenic properties of foodstuffs. In traditional oriental medicine, various local herbs and animal products remain actively utilized for the medicine. Apitherapy (honey bee venom therapy) is a good example of the differences.

These differences in culprit allergens can influence the efficacy of immunotherapy as well as diagnostic sensitivity. However, diagnosis and immunotherapy in Asia rely on the allergen extracts produced from western countries. Some allergens of regional importance are not yet commercially available, and allergy diagnoses depends on cross-reactivity with the extracts from similar species. Furthermore, edible insect allergens should be taken into account since some studies are being performed to develop future diet and animal feed without sufficient investigations on allergic side effects.

This review summarizes the important species in Korea and their cross-reactivity with the representative species. Allergen characteristics will also be discussed if molecular studies have been completed.

## Inhalant Allergens of Regional Importance

The 10 most common inhalant allergens (*D. farinae, D. pteronyssinus, Tyrophagus putrescentiae*, cat epithelium, birch, mugwort, alder, hazel, beech, and oak) account for 90% of inhalant allergen sensitization in a retrospective analysis of skin tests (1). However, many of the positives are not of primary sensitization, but as a result of cross-reactions. A significant increase of skin reactivity over 30 years in Korea was observed with oak (4.7 to 14.4%), birch (7.1 to 13.6%), alder (6.3 to 13.4%), and pine (2.9 to 14.3%) in the 2010s compared to the 1990s (2). Skin reactivity to grass (13.9 to 20.3%) and weed (27.0 to 40.9%) pollens increased, while no differences were observed with house dust mites (55.2 to 55.6%) during the same period.

### Tree Pollen

Trees in the spring and weeds in autumn are is the most important causes of pollinosis in Korea. The pollen concentrations of oak in the spring and Japanese hop during the autumn season were the most common in the Korean atmosphere (3). Currently, no pollen extracts from oak and birch native to Korea are commercially available (Table 1). Siberian silver birch, instead of common silver birch, is native to Korea. However, birch trees remain uncommon. The most abundant oak species in Korea is the Mongolian oak. Que m 1 from Mongolian oak exhibited better diagnostic value than Bet v 1 (4). However, most Mongolian oaks are found in the mountains with sawtooth oak being common near villages. Moreover, sawtooth oak pollen extracts exhibited stronger allergenic activity compared to Mongolian oak pollen extract (5). Recently, the allergenicity of Que ac 1 from sawtooth oak was investigated (Jeong et al. manuscript in submission). Que ac 1 was shown to be highly polymorphic. The IgE reactivity of recombinant Que ac 1 was more potent than Bet v 1 in Korean oak pollinosis patients. However, no significant differences were displayed in mediator release assays with rat basophilic leukemia cells.

**Table 1:** Comparison of native and imported commercial plant species for clinical use.

**Plant**	**Imported species for clinical use**		**Native species**	
	**Scientific name**	**Common name**	**Scientific name**	**Common name**
Birch	*Betula pendula*	Common silver birch	*Betula platyphylla* var. *japonica*	Siberian/Japanese silver birch
Oak	*Quercus alba*	White oak	*Q. mongolica*	Mongolian oak
			*Q. acutissima*	Sawtooth oak
			*Q. dentata*	Japanese emperor/Daimyo oak
			*Q. aliena*	Oriental white oak
			*Q. variabilis*	Chinese cork oak
Ragweed	*Ambrosia artemisiifolia*	Common/short ragweed	*Ambrosia artemisiifolia*	Common/short ragweed
Mugwort	*Artemisia vulgaris*	Mugwort	*Artemisa princeps*	Korean mugwort
Hop	*Humulus lupulus*	Common hop	*Humulus japonicus*	Japanese hop

### Weed Pollen

#### Japanese Hop

Autumn pollinosis in Korea is elicited by weed species like ragweed, mugwort, and Japanese hop. Grass causes summer pollinosis, but is rare in Korea. Japanese hop is of particular interest for its high atmospheric concentration in East Asia and recent invasion into Western countries. Interestingly, it exhibited no essential cross-reactivity with a common hop, a closely related species, nor mugwort or ragweed (6–8). However, the molecular details of its allergens have yet to be characterized. A 10 kDa component is known to be a major allergen (7) even though it has not been cloned. Recombinant proteins of profilin, pathogenesis-related 1, polygalacturonase, and pectin methyl esterase, homologous to pollen allergens from Japanese hop, were recognized with 3.4 to 13.8% of IgE antibodies from the patients (9, 10).

#### Mugwort

Up to 500 different mugwort species have been described worldwide (11). In Korea, 26 mugwort species have been recorded with *Artemisia princeps* being the most prevalent (12). Fortunately, a high sequence identity of three groups of allergens (Art v 1, 2, and 3) from different species and almost equivalent IgE binding capacity of these allergens were reported, allowing diagnosis and immunotherapy with commercialized mugwort extracts (13, 14).

### House Dust Mites and Spider Mites

House dust mites are the most frequent cause of allergic diseases (15). Cross-reactivity between house dust mites and storage mites often lead to false-positive reactions to storage mites (16, 17). Polymorphisms of the major allergens from Korean house dust mite isolates (18) were investigated, and sensitization patterns to component allergens were also examined (19, 20). Mite allergic patients suffering from respiratory symptoms were more likely to be sensitized only to Der f 1 and Der f 2 allergens. However, patients suffering from cutaneous symptoms were shown to be sensitized to minor allergens, Der f 11, Der f 13, Der f 14, Der f 32, and Der f Alt a 10 (20). Notably, further studies on IgE reactivity to some of component allergens such as Der p 23, known as a major allergen from Western countries (21), are to be done. Recombinant Der f 23 was recognized by 42.8% of serum IgE from patients, while Der f 2 was recognized by 96.4% (22).

More interestingly, a high sensitization rate to spider mites was described (23). However, the molecular characterization of its allergens has not completed. Cross-reactivity between spider mites and house dust mites should also be performed to verify whether primary sensitization to spider mites is common.

### Midges

It is possible that allergic reactions to swarming insect species such as mayflies, stoneflies, and midges can occur. Hemoglobin-like proteins, which help with oxygen uptake in the water, have long been described as the major allergen from the blood worm, chironomid larvae (24). However, these molecules are not found in the adult stage. Tropomyosin, a highly cross-reactive invertebrate pan-allergen, was described as a major allergen from the adult midge of a dominant species (25). A 42 kDa protein was recently identified as a novel allergen from *Cricotopus bicintus*, a hemoglobin-free midge (26).

### Companion Animals

Certain dog breeds are being marketed as being hypoallergenic without reliable scientific evidence (27), and some recent studies suggest no evidence of hypoallergenic dog breeds (27, 28). However, the possible different allergenicity of different dog breeds has been described (29–31). Therefore, more investigations are needed on small-sized dog breeds such as the Maltese, Pomeranian, and Poodle, which are popular in Korea. Genetic differences and living environments, including housing situation, diet, and washing habits, may influence the production and accumulation of allergenic substances.

## Food Allergens of Regional Importance

Egg and milk are the most common causes of allergic diseases in Korean children (32), while fruits associated with pollen food allergy syndromes, wheat, and crustaceans are the most frequent causative allergens in Korean adult subjects (33, 34). Interestingly, sensitization to silkworm pupa was most common (25.4%), but the majority of the silkworm pupa-sensitized subjects were asymptomatic to exposure.

### Fruits and Food Allergy

Foods (84.8%) are the most common cause of anaphylaxis, followed by drugs (7.2%) in Korean children (35), while drugs (58.3%) are the most common cause, followed by food (28.3%) in adults. The major causative foods of immediate-type food allergy were cow's milk (28.1%), hen's eggs (27.6%), wheat (7.9%), walnut (7.3%), peanut (5.3%), buckwheat (1.9%), and shrimps (1.9%) in children (36). Among the Korean PFAS patients, 8.9% suffered from anaphylaxis (37). The most common cause of anaphylaxis in PFAS was peanut (33.3%), followed by apple (33.3%), walnut (22.2%), pine nut (18.5%), peach (14.8%), and ginseng (14.8%) (38). Foods associated with PFAS are peach (48.5%), apple (46.7%), kiwi (30.4%), peanut (17.4%), plum (16.3%), chestnut (14.8%), pineapple (13.7%), walnut (14.1%), Korean melon (12.6%), tomato (11.9%), melon (11.5%), apricot (10.7%). Interestingly, Korean foods such as taro (8.9%), ginseng (8.2%), sesame leaf (4.4%), bellflower root (4.4%), crown daisy (3.0%), deodeok (3.3%), kudzu root (3.0%) and lotus root (2.6%) also cause PFAS (36). The studies on sensitization pattern to component allergens can provide better picture of cross-reactivity and peculiarity of Korean foods. However, molecular characterizations of major allergens from Korean foods have not been done.

### Buckwheat

Buckwheat is a leading cause of anaphylaxis in Korea (39). Various components (9, 16, 19, and 24 kDa) are IgE reactive (40). Among these components, a 16 kDa 2S albumin designated Fag e 2 (41), and a 19 kDa vicilin-like protein designated Fag e 3 (42) are shown to be frequently recognized by IgE from symptomatic allergic patients. However, a 24 kDa protein (Fag e 1), 13 S globulin seed storage protein 3, and legumin are also recognized by asymptomatic sensitized individuals. Recently, a 3.9 kDa antimicrobial peptide designated Fag e 4 and a 55 kDa vicilin-like protein designated Fag e 5 were characterized (43). However, more studies are necessary since only a small number of patients (*n* = 7) were tested for Fag e 4 (5/7) and Fag e 5 (6/7) allergens. Furthermore, Fag e 4 is homologous to hevein and may possibly be cross-reactive with latex allergens.

### Fish

Chub mackerels, pollacks, largehead hairtails, redlip croakers, flounders, eels, and anchovies are the most commonly consumed fishes in Korea. Interestingly, a very limited number of fish species, such as codfish and mackerel, are currently utilized to diagnose fish allergies in Korea (Table 2). No studies in Korea have been performed into the molecular details of fish allergens. A recent study showed that parvalbumin is the single most potent allergen in Korea, and extensive cross-reactivity among fish species is reported (In press). The high cross-reactivity of parvalbumin allows diagnosis with sibling species. Still, it possible to be sensitized to minor allergens which are not cross-reactive. Therefore, it may be necessary to look into the minor allergens from various fish species in greater detail.

**Table 2:** Comparison of native and imported commercial fish species for clinical use.

**Fish**	**Imported species for clinical use**		**Native species**	
	**Scientific name**	**Common name**	**Scientific name**	**Common name**
Cod	*Gadus morhua*	Atlantic cod	*Gadus macrocephalus*	Pacific cod
Mackerel	*Scomber scombus* *Scomber japonicus* *Scomber australasicus*	Atlantic mackerel Chub mackerel Japanese/Pacific mackerel	*Scomber japonicus*	Chub mackerel
Cutlass	Not available		*Trichiurus lepturus*	Largehead hairtail
Croaker	Not available		*Larimichthys polyactis*	Redlip croaker
Anchovy	*Engraulis* spp.		*Engraulis japonicus*	Japanese anchovy

### Edible Insects

The edible insect industry is rapidly growing to overcome food shortages associated with population growth (44). However, more attention should be given to possible allergic adverse reactions after the ingestion of edible insects (45).

The Korean government recently created a code for the edible insect industry. In this code, mealworms, silkworms, Rice grasshoppers, rhinoceros beetles, white-spotted flower chafers, and two-spotted crickets are classified as edible insects (Table 3). More studies must be performed on mealworm allergens, which are the most frequently consumed insect worldwide. The most commonly identified IgE reactive molecules are tropomyosin, arginine kinase, paramyosin, chitinase, α-amylase, and hexamerin (46, 49–52).

**Table 3:** Edible insects and their potential component allergens.

**Insect**	**Component allergen**			
**Common name**	**Scientific name**	**Protein**	**IUIS nomenclature**	**Reference**
Silkworm (pupa)	*Bombyx mori*	Arginine kinase 27 kDa glycoprotein Tropomyosin Paramyosin Chitinase	Bomb m 1	Liu et al. (46) Jeong et al. (47) Jeong et al. (48) Zhao et al. (49) Zhao et al. (49)
Mealworm (larva)	*Tenebrio molitor*	Arginine kinase Tropomyosin α-amylase Paramyosin Hexamerin/hemocyanin Myosin Trypsin Serine protease		Verhoeckx et al. (50) Verhoeckx et al. (50) Verhoeckx et al. (50) van Broekhoven et al. (51) van Broekhoven et al. (51) van Broekhoven et al. (51) Verhoeckx et al. (50) Verhoeckx et al. (50)
White-spotted flower chafer (larva)	*Protaetia brevitarsis seulensis*			
Two-spotted cricket	*Gryllus bimaculatus*	Arginine kinase Hexamerin/hemocyanin		Srinroch et al. (52) Srinroch et al. (52)
Rice grasshopper	*Oxya chinensis sinuosa*			
Rhinoceros beetle (larva)	*Allomyrina dichotoma*			

As mentioned above, false-positive reactions (asymptomatic sensitization) to silkworm pupa remain an unsolved problem for Korean allergic subjects (33). Many studies have focused on cross-reactivity by tropomyosin, mainly from shrimp- and house dust mite-allergic patients, to edible insects. However, silkworm tropomyosin does not have strong allergenicity in Korean patients (48). We identified a 27-kDa glycoprotein as a heat-stable allergen (47) and are working on the characterization of more silkworm pupa allergens.

## Miscellaneous Allergens

### Stinging Insects

Honey bees, bumble bees, yellow jackets, and hornets are the most frequent causes of insect sting anaphylaxes. They are highly cross-reactive with imported stinging insects even though many of them are different species. The Asian needle ant is of particular interest because it is also regarded as an invasive species to western countries. Pac c 3, antigen 5, is the most important allergenic component as found in other wasps (53). However, it is only partially cross-reactive with Ves v 5, a homologous allergen from yellow jackets (54).

## Allergen Standardization in Korea

In Korea, studies on allergen standardization have been performed with support from the Korean Center for Disease Control and Prevention since 2009 (55, 56). Standardization for the extracts from house dust mites (57), cockroaches (58), and some pollens (mugwort and Japanese hop) (59) was performed and now food allergen standardization is being conducted. Two-site ELISA systems for the quantification of buckwheat allergen Fag e 3 (60) and house dust mite allergen Der f 1 (In press) have been developed. A company with a good manufacturing practice facility for the production of allergen extracts for allergy immunotherapy was established in 2017 (61).

## Perspectives for the Development of Better Allergy Diagnostics

Challenge test is the most accurate choice of diagnosis, especially for food allergy. However, physicians should take risks of possible adverse reaction including anaphylaxis. Component-resolved diagnosis (CRD) with recombinant or native allergens could be useful for the identification of primary sensitizers and more accurate diagnosis of allergic diseases (62). CRD can discriminate true food allergy from cross-reactive hypersensitivity in food allergy patients, such as pollen food allergy syndrome (63).

Mediator release from effective cells (mast cell and basophil) is dependent not only on the avidity of IgE antibodies to an allergen, but also on the ability to cross-link FcεR1 and to compete with blocking antibodies (64). The distance between two different IgE epitopes may have an influence on the immune complex shape and aggregation of FcεRI and thus the subsequent activation of basophils (65). Some allergens are not recognized due to its low abundance in the allergenic sources and its extracts. Furthermore, cross-reactive carbohydrate determinant (CCD) often causes false-positive IgE reactions without clinical manifestation (66). Basophil activation test (BAT) could be utilized to overcome these limitations. Some Korean scientists carry out BAT for research purpose, but BAT is rarely utilized in clinical fields. Development of more convenient BAT kit may needed for more common use in clinics.

## Author Contributions

Conceptualization and writing of the original draft, and final approval by both authors.

## Conflict of Interest

The authors declare that the research was conducted in the absence of any commercial or financial relationships that could be construed as a potential conflict of interest.
